# Liquiritin modulates ERK- and AKT/GSK-3β-dependent pathways to protect against glutamate-induced cell damage in differentiated PC12 cells

**DOI:** 10.3892/mmr.2014.2289

**Published:** 2014-05-30

**Authors:** LESHENG TENG, QINGFAN MENG, JIAHUI LU, JING XIE, ZHENZUO WANG, YAN LIU, DI WANG

**Affiliations:** 1 College of Life Science, Jilin University, Changchun, Jilin 130012, P.R. China; 2 Key Laboratory for Molecular Enzymology and Engineering of the Ministry of Education, Jilin University, Changchun, Jilin 130021, P.R. China

**Keywords:** liquiritin, neuroprotection, glutamate, extracellular signal-regulated kinases, AKT/glycogen synthase kinase-3β

## Abstract

Glutamate has a key role in the neuronal cell damage associated with Alzheimer’s and Parkinson’s diseases. Liquiritin (LQ), a major constituent of Glycyrrhiza Radix, possesses various pharmacological activities. The present study investigated the neuroprotective effect of LQ against glutamate-induced cell damage in the differentiated PC12 (DPC12) rat pheochromocytoma cell line. Pretreatment with 25 and 50 μM LQ for 3 h resulted in a significant increase in cell viability and inhibited excessive lactate dehydrogenase release in glutamate-exposed DPC12 cells. LQ also ameliorated glutamate-induced nuclear and mitochondrial apoptotic alterations, intracellular calcium overload and the abnormal expression of apoptosis-related proteins, including cytochrome *c*, B-cell lymphoma (Bcl)-2 and Bcl2-associated X protein. Treatment with LQ alone or in combination with glutamate was found to enhance the phosphoactivation of extracellular signal-regulated kinases (ERKs), AKT and its downstream element glycogen synthase kinase-3β (GSK3β), in a time-dependent manner. However, no effect was observed on the expression of total-ERKs, -AKT and -GSK3β. Furthermore, pre-incubation with 10 μM PD98059 or LY94002, inhibitors of ERK and phosphatidylinositide 3-kinase, respectively, for 30 min significantly suppressed the LQ-induced increase in glutamate-exposed DPC12 cell viability. To the best of our knowledge, the present study provides the first experimental evidence that LQ has a neuroprotective effect against glutamate toxicity in DPC12 cells, predominantly through the ERK and AKT/GSK-3β pathways. Therefore, LQ may have potential for the treatment of neurodegenerative diseases.

## Introduction

**Figure 1:**
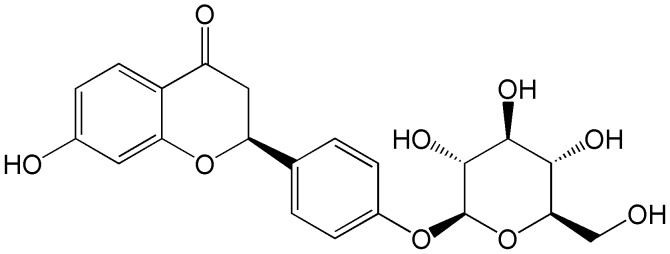
Chemical structure of liquiritin.

Glycyrrhiza Radix has been used as a treatment for thousands of years in China and its major components have been reported to exhibit various pharmacological activities, including anti-inflammatory (1), -obesity (2), -viral (3), -oxidative (4) and neuroprotective (5) effects. Liquiritin (LQ), one of the major compounds extracted from Glycyrrhiza Radix, possesses anti-depressant-like effects, as has been indicated by tail-suspension and forced swimming tests in mice (6). LQ also exerts neurotrophic effects, whereby it promotes nerve growth factor (NGF)-induced neurite outgrowth (7). The chemical structure of LQ is shown in Fig. 1. A previous study has reported that LQ may exert neuroprotective effects in cerebral ischemia/reperfusion-induced brain damage through antioxidant and anti-apoptotic mechanisms (8). However, the neuroprotective effect of LQ against glutamate-induced cell damage has not yet been elucidated.

Glutamate, an important neurotransmitter in the vertebrate nervous system, has a key role in learning and memory (9). Glutamate-mediated excitotoxicity occurs as part of the ischemic cascade (10) and is associated with numerous diseases, including amyotrophic lateral sclerosis, autism, Alzheimer’s disease and certain forms of mental retardation (9). Several signaling pathways are involved in the regulation of glutamate-induced neurotoxicity (11,12). Extracellular signal-regulated kinases (ERKs) and AKT signaling pathways have been proposed to contribute to cell differentiation, proliferation, survival and apoptosis (13–15). Furthermore, previous studies have demonstrated that glutamate significantly downregulates AKT and ERK phosphorylation (16,17). A previous study has also shown that sodium ferulate protects cortical neurons against glutamate-induced apoptosis through phosphatidylinositide 3-kinase (PI3K)/AKT and ERK signaling pathways (17).

In the present study, LQ was found to protect differentiated PC12 (DPC12) cells against glutamate-induced reduced cell viability, high apoptosis rates, excessive lactate dehydrogenase (LDH) release, intracellular Ca^2+^ overload and mitochondrial dysfunction. Furthermore, LQ pretreatment was observed to normalize the glutamate-induced alterations in pro- and anti-apoptotic protein expression. The LQ-mediated neuroprotective effect against glutamate-induced DPC12 cell damage was found to be associated with ERK and AKT activation.

## Materials and methods

### Cell lines and culture

PC12 cells (CRL-1721; American Type Culture Collection, Rockville, MD, USA) were used at passages <10 and were maintained as monolayer cultures in Dulbecco’s Modified Eagle Medium (DMEM) supplemented with 10% horse serum (HS; Invitrogen Life Technologies, Carlsbad, CA, USA), 5% fetal bovine serum (FBS; Invitrogen Life Technologies), 100 U/ml penicillin and 100 μg/ml streptomycin in a humidified atmosphere containing 5% CO_2_ and 95% air at 37°C. Cells were differentiated using the addition of 20 ng/ml NGF (Sigma-Aldrich, St. Louis, MO, USA) in DMEM supplemented with 1% HS, 1% FBS, 100 U/ml penicillin and 100 μg/ml streptomycin for 48 h.

### Cell viability assay

Cell viability was measured using a quantitative colorimetric assay with MTT (Sigma-Aldrich) as described previously (18). Briefly, PC12 cells were seeded onto 96-well plates at a density of 2×10^4^/well and differentiated using NGF. Cells were pretreated with 25 and 50 μM LQ (purity >98.0%; Shanghai Source Leaves Biological Technology Co., Ltd., Shanghai, China) for 3 h and co-treated with 20 mM glutamate for 24 h. In separate experiments, DPC12 cells underwent 30 min pretreatment with 10 μM PD98059, an ERK inhibitor, or 10 μM LY294002, a PI3K inhibitor. Cells were then treated with 25 or 50 μM LQ for 3 h, prior to exposure to 20 mM glutamate for 24 h. Treated cells were subsequently incubated with MTT solution (0.5 mg/ml) for 4 h at 37°C in the dark. The absorbance was measured using a microplate reader (Bio-Rad Laboratories, Inc., Hercules, CA, USA) at 540 nm. The viability of the treated cells was expressed as a percentage of that of the corresponding control cells.

### Released LDH analysis

The *In Vitro* Toxicology Assay kit (Sigma-Aldrich) was used to detect LDH release in the culture medium. PC12 cells were seeded onto six-well plates at a density of 1×10^5^/well and were differentiated using NGF. DPC12 cells were pretreated with 25 and 50 μM LQ for 3 h and then co-treated with 20 mM glutamate for 24 h. The medium in each treatment group was collected individually. A total of 60 μl mixed assay solution was added to 30 μl culture medium. Following incubation at room temperature in the dark for 30 min, 10 μl 1 N HCl was added to terminate the reaction. Absorbance was spectrophotometrically measured at a wavelength of 490 nm. LDH release in the treatment groups was expressed as a percentage of the LDH released in the control group.

### Flow cytometric analysis of apoptosis

Annexin V and propidium iodide (PI) double staining was used to determine alterations in cell apoptosis. PC12 cells were seeded onto six-well plates at a density of 1×10^5^/well and differentiated. DPC12 cells were then pretreated with 25 and 50 μM LQ for 3 h, prior to co-treatment with 20 mM glutamate for 24 h. Subsequent to collection, cells were suspended in binding buffer containing 20 μg/ml Annexin V-fluorescein isothiocyanate and 50 μg/ml PI, and incubated for 20 min at room temperature. Cell apoptosis rate was analyzed using a flow cytometer (FC500; Beckman Coulter, Inc., Brea, CA, USA).

### Intracellular Ca^2+^ concentration analysis

Cells were stained with Fluo-4 AM (Invitrogen Life Technologies) at a final concentration of 5 μM in order to determine the intracellular Ca^2+^ concentration. PC12 cells were seeded onto confocal dishes at a density of 1×10^5^ cells/well and differentiated. Subsequent to pretreatment with 25 μM LQ for 3 h and co-treatment with 20 mM glutamate for 12 h, cells were incubated with Fluo-4 AM for 30 min at 37°C in the dark. Following three washes with phosphate-buffered saline (PBS), the fluorescence intensity was determined using laser scanning confocal microscopy (Axio Observer Z1; Carl Zeiss, Oberkochen, Germany) with an excitation wavelength of 488 nm and an emission wavelength of 520 nm at a magnification of ×20.

### Mitochondrial membrane potential (Δψm) analysis

5,5′,6,6′-Tetrachloro-1,1′,3,3′ tetraethylbenzimidazolylcarbocyanine iodide (JC-1; Sigma-Aldrich) staining was used to examine alterations in Δψm. PC12 cells were seeded onto confocal dishes at a density of 1×10^5^ cells/well and differentiated. Subsequent to pretreatment with 25 μM LQ for 3 h and co-treatment with 20 mM glutamate for 12 h, cells were incubated with 2 μM JC-1 at 37°C for 10 min in the dark. Following three washes with PBS, changes in mitochondrial fluorescence were examined using a fluorescent microscope (Axio Observer Z1; Carl Zeiss) at a magnification of ×20. Red fluorescence was observed in healthy cells with a high Δψm and green fluorescence was apparent in apoptotic or unhealthy cells with a low Δψm (19).

### Western blot analysis

Treated cells were lysed in radioimmunoprecipitation assay buffer containing 1% protease inhibitor cocktail and 2% phenylmethanesulfonyl fluoride (Sigma-Aldrich). In order to detect cytochrome *c* (cyto *c*) release, cytoplasmic extracts were prepared as described previously by Yang *et al* (20). A total of 30 μg protein was separated using 10–12% SDS-PAGE and electrophoretically transferred onto nitrocellulose membranes (pore size, 0.45 μm; Bio Basic, Inc., Markham, ON, Canada). The transferred membranes were then blotted with antibodies against phosphorylated (P)-ERKs, total (T)-ERKs, P-AKT, T-AKT, P-glycogen synthase kinase-3β (GSK3β), T-GSK3β, B-cell lymphoma 2 (Bcl-2), Bcl2-associated X protein (Bax), cyto *c* and GAPDH at dilutions of 1:1,000 (Cell Signaling Technology, Inc., Danvers, MA, USA) at 4°C overnight. Membranes were then incubated with horseradish peroxidase-conjugated secondary antibodies (Santa Cruz Biotechnology, Inc., Santa Cruz, CA, USA) for 3 h at 4°C. Chemiluminescence was detected using enhanced chemiluminescence detection kits (GE Healthcare, Amersham, UK). The intensity of the bands was quantified by scanning densitometry using Quantity One 4.5.0 software (Bio-Rad Laboratories, Inc.).

### Statistical analysis

One-way analysis of variance was used to detect statistical significance, followed by post hoc multiple comparison tests. Data are expressed as the mean ± standard deviation. A value of P<0.05 was considered to indicate a statistically significant difference.

## Results

### LQ protects DPC12 cells from glutamate-induced apoptotic cell damage

**Figure 2:**
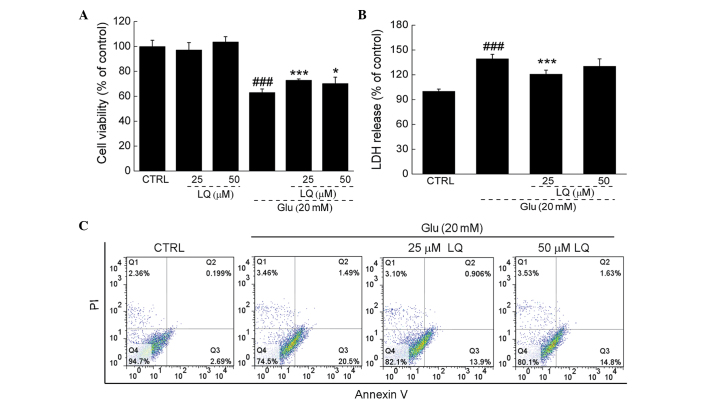
LQ has a protective effect against Glu-induced apoptosis in DPC12 cells. Cells were pretreated with 25 and 50 μM LQ for 3 h, followed by exposure to 20 mM Glu for 24 h. (A) LQ significantly enhanced cell viability compared with Glu-treated cells. (B) LQ reduced excessive Glu-induced LDH release in DPC12 cells. (C) LQ downregulated the apoptosis rate in Glu-exposed DPC12 cells. Data are expressed as a percentage of the value in the control group and presented as the mean ± standard deviation of three replicate experiments. ^###^P<0.001 vs. control group; ^*^P<0.05 and ^***^P<0.001 vs. Glu-treated cells. LQ, liquiritin; LDH, lactate dehydrogenase; Glu, glutamate; PI, propidium iodide; CTRL, control.

Exposure of DPC12 cells to 20 mM glutamate for 24 h resulted in ~38% cell death; however, upon pretreatment with 25 or 50 μM LQ for 3 h, cell death was significantly reduced (71 and 74% viability vs. 62% viability, P<0.05). Pretreatment with 25 and 50 μM LQ alone showed no effect on cell proliferation (Fig. 2A).

In DPC12 cells exposed to 20 mM glutamate, LDH release was observed to be 39% greater than that in the control cells (P<0.001). However, pretreatment with 25 μM LQ was found to significantly suppress LDH release to levels 20% higher than those in the control cells (139 vs. 120%, P<0.001) (Fig. 2B). Furthermore, flow cytometry revealed that LQ reduced the proportion of apoptotic cells compared with the cells solely exposed to glutamate (Fig. 2C).

### LQ attenuates intracellular Ca^2+^ overload and restores the dissipation of Δψm

**Figure 3:**
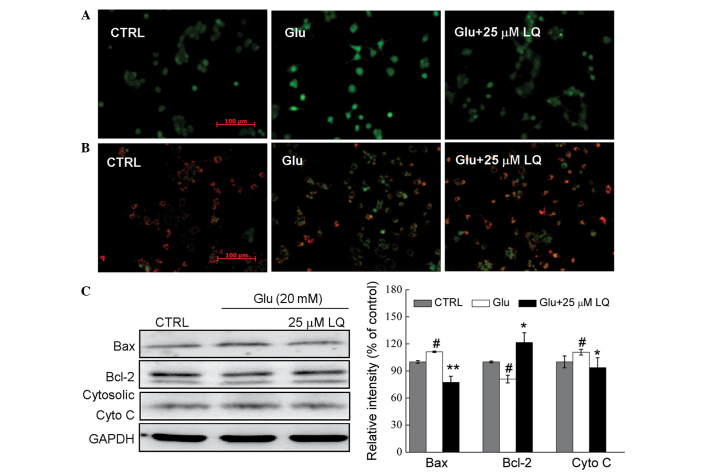
In Glu-exposed DPC12 cells, LQ restores (A) intracellular Ca^2+^ overload (magnification, ×20), (B) mitochondrial membrane potential dissipation (magnification, ×20) and (C) alterations in the expression of apoptosis-related proteins. Cells were pretreated with 25 μM LQ for 3 h and exposed to 20 mM Glu for (A and B) 12 h or (C) 24 h. Bcl-2, Bax and cytosolic cyto *c* expression was normalized using GAPDH. Data are expressed as a percentage of the value in the corresponding control group and are presented as the mean ± standard deviation of three replicate experiments. ^#^P<0.05 vs. control group; ^*^P<0.05 and ^**^P<0.01 vs. Glu-treated cells. LQ, liquiritin; Glu, glutamate; Bcl-2, B-cell lymphoma-2; Bax, Bcl2-associated X protein; cyto *c*; cytochrome *c*; CTRL, control.

Fluo-4 AM staining was used to assess the changes in Ca^2+^ concentration in DPC12 cells. In cells exposed to 20 mM glutamate for 12 h, high Ca^2+^ influx was observed, as indicated by the increase in fluorescence intensity. Pretreatment with 25 μM LQ was found to reduce this Ca^2+^ overload (Fig. 3A).

Mitochondrial function is one of the factors responsible for cell apoptosis. JC-1 staining revealed that pretreatment with 25 μM LQ (21) significantly restored the glutamate-induced dissipation of Δψm, as indicated by an increase in red fluorescence in the LQ-pretreated cells compared with those treated solely with glutamate (Fig. 3B).

Glutamate exposure was found to enhance Bax expression by 11%, reduce Bcl-2 expression by 20% and increase cytosolic cyto *c* expression by 10% compared with the non-treated control cells (all P<0.05). However, LQ markedly reduced the glutamate-induced increase in Bax and cytosolic cyto *c* expression to normal levels, and enhanced the expression of Bcl-2 (P<0.05) (Fig. 3C).

### ERK and AKT/GSK3β activation contributes to LQ-mediated neuroprotection in DPC12 cells

**Figure 4:**
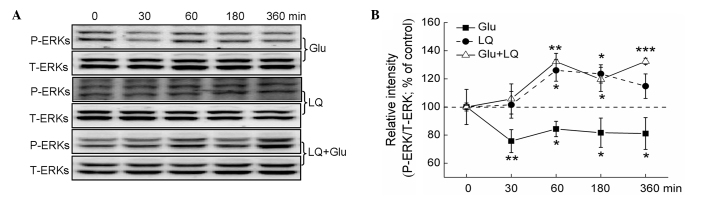
ERK pathways are involved in LQ-mediated neuroprotection against Glu-induced cell damage. DPC12 cells were treated with LQ or Glu alone and collected at 0, 30, 60, 180 and 360 min. For LQ and Glu co-treatment, DPC12 cells were pretreated with 25 μM LQ for 3 h, followed by Glu. Cells were then collected at 0, 30, 60, 180 and 360 min subsequent to Glu exposure (A) Expression of P-ERKs and T-ERKs detected using western blot analysis. (B) Quantification of the expression of P-ERKs and T-ERKs. The expression of P-ERKs was normalized using that of T-ERKs. Data are presented as the mean ± standard deviation of three replicate experiments. ^*^P<0.05, ^**^P<0.01 and ^***^P<0.001 vs. cells collected at 0 min. ERK, extracellular signal-regulated kinase; Glu, glutamate; LQ, liquiritin; P-, phosphorylated; T-, total.

ERK and AKT/GSK3β activation was detected in DPC12 cells. While glutamate exposure for between 30 and 360 min was found to significantly inhibit ERK phosphorylation, exposure to 25 μM LQ alone for 60 and 180 min was found to significantly enhance the expression of P-ERKs (P<0.05). Furthermore, pretreatment with LQ for between 60 and 360 min was observed to significantly reverse the glutamate-induced suppression of P-ERK expression (P<0.05) (Fig. 4A and B).

**Figure 5:**
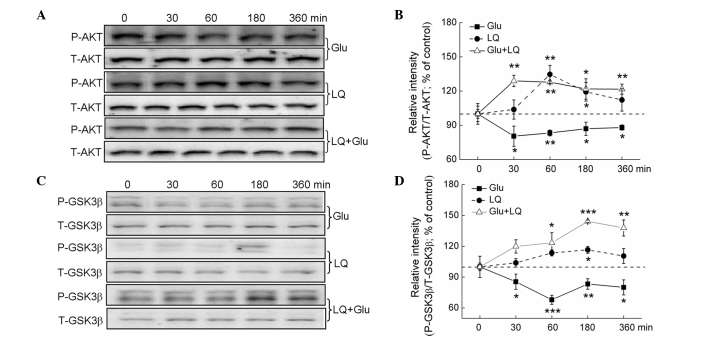
The AKT/GSK3β pathway contributes to LQ-mediated neuroprotection against Glu-induced cell damage. DPC12 cells were treated with LQ or Glu alone and collected at 0, 30, 60, 180 and 360 min. For LQ and Glu co-treatment, DPC12 cells were pretreated with 25 μM LQ for 3 h, followed by Glu. Cells were then collected at 0, 30, 60, 180 and 360 min subsequent to Glu exposure. (A and C) Expression of P-AKT, T-AKT, P-GSK3β and T-GSK3β detected using western blot analysis. (B and D) Quantification of P-AKT and P-GSK3β expression, normalized using T-AKT and T-GSK-3β expression, respectively. Data are expressed as a percentage of the value in the corresponding control group and presented as the mean ± standard deviation of three replicate experiments. ^*^P<0.05, ^**^P<0.01 and ^***^P<0.001 vs. cells collected at 0 min. GSK3β, glycogen synthase kinase-3β; P-, phosphorylated; T-, total; LQ, liquiritin; Glu, glutamate.

PI3K/AKT are crucial regulators of glutamate-mediated cell damage (17). Glutamate treatment for between 30 and 360 min was found to significantly suppress P-AKT and P-GSK3β expression. Exposure to LQ alone and in combination with glutamate resulted in a time-dependent increase in P-AKT and P-GSK3β expression (P<0.05), but did not affect expression of T-AKT and T-GSK3β (Fig. 5A–D).

**Figure 6:**
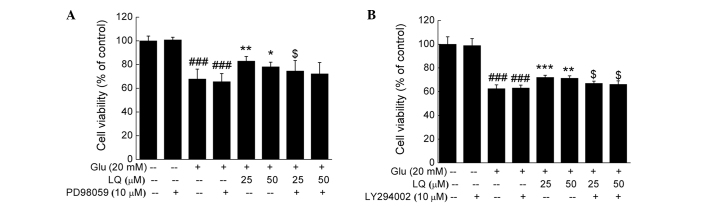
PD98065 and LY294002 partially abolish the enhanced cell viability induced by LQ. Cells were pretreated with (A) 10 μM PD98065 or (B) 10 μM LY294002 for 30 min, followed by treatment with 25 and 50 μM LQ for 3 h and exposure to 20 mM Glu for 24 h. Data are expressed as a percentage of the cell viability of the corresponding control group and are presented as the mean ± standard deviation of three replicate experiments. ^*^P<0.05, ^**^P<0.01 and ^***^P<0.001 vs. control cells; ^###^P<0.001 vs. Glu-treated cells; ^$^P<0.05 vs. LQ plus Glu-treated cells. LQ, liquiritin; Glu, glutamate.

DPC12 cells underwent 30 min pretreatment with 10 μM ERK or PI3K inhibitor, PD98059 or LY294002 respectively, followed by 3 h treatment with LQ and 24 h exposure to glutamate. Treatment with PD98059 or LY294002 did not affect cell viability compared with the untreated or glutamate-treated cells; however, it was found to significantly reduce the potency of LQ in enhancing cell viability (P<0.05) (Fig. 6).

## Discussion

The present study investigated the neuroprotective effect of LQ against glutamate-induced cell damage and its underlying mechanism. LQ was found to significantly attenuate the glutamate-induced decrease in DPC12 cell viability and apoptotic alterations, including mitochondrial function, the expression of apoptosis-related proteins, intracellular Ca^2+^ concentration and LDH release. Furthermore, the activation of ERKs and AKT/GSK-3β was found to contribute to LQ-mediated neuroprotection.

Dissipation of Δψm and elevated mitochondrial cyto *c* release were observed in glutamate-exposed DPC12 cells. Experimental evidence has indicated that mitochondria have a key role in executing important intracellular events associated with neuronal survival and apoptosis (21). Certain apoptosis-related proteins, including Bcl-2 and Bax, target the mitochondria and induce mitochondrial swelling or increase the permeability of the mitochondrial membrane. This leads to the efflux of apoptotic effectors from the mitochondria (22,23). Cyto *c*, released from mitochondria, serves as a regulatory factor in morphological apoptosis-related changes (24). In the present study, after 3 h pretreatment with LQ, the glutamate-induced dissipation of Δψm was markedly restored and the expression of Bcl-2, Bax and cytosolic cyto *c* was normalized. These findings indicate that the neuroprotective effect of LQ may, at least partly, be attributed to its restoration of Δψm through upregulation of the activity of mitochondria-dependent apoptotic molecules.

AKT activation is associated with cell survival and proliferation (25). GSK-3β, a constitutively active enzyme substrate of AKT, is inactivated by P-AKT (26). It has been reported that GSK-3β inactivation is involved in the guanosine-mediated protective effects against glutamate-induced cell death in SH-SY5Y cells (26). Furthermore, GSK-3β inhibition has been found to protect against ischemia/reperfusion organ injury (27). In the present study, exposure to LQ alone or in combination with glutamate was observed to markedly enhance P-AKT and P-GSK3β levels in a time-dependent manner in DPC12 cells compared with untreated cells. In addition, pretreatment with the PI3K/AKT inhibitor LY294002 was found to partially antagonize the LQ-induced increase in cell viability. Furthermore, the increase in AKT activation observed upon pretreatment with LQ resulted in an increase in GSK3β phosphorylation, which has an important role in LQ-mediated neuroprotection. Previous studies have suggested that the activation of AKT regulates the expression of Bcl-2 (28). The AKT/Bcl-2 pathway contributes to the protective effect of sodium ferulate in cultured cortical neurons (17). Bcl-2 acts as an upstream checkpoint of mitochondrial function (29); therefore, the findings of the present study may indicate that mitochondrial function is associated with AKT activation in LQ-exposed DPC12 cells.

ERKs were also analyzed in the present study. Treatment with LQ alone or in combination with glutamate was found to induce rapid phosphorylation of ERKs, whereas glutamate treatment alone was observed to reduce P-ERK expression. PD98059 diminished the protective effect of LQ against the glutamate-induced neurotoxicity and reduction in cell viability. It has previously been reported that the inhibition of ERKs using a specific inhibitor results in downregulation of Bcl-2 (30). These findings suggest that the protective effect mediated by LQ may be achieved through ERK pathways, which may be associated with mitochondrial function.

In conclusion, to the best of our knowledge, the present study provides the first experimental evidence that LQ has a neuroprotective effect against glutamate-induced cell damage, and that this effect is associated with ERK and AKT/GSK3β pathways in DPC12 cells. These findings suggest that LQ may have potential as a therapeutic agent for the treatment of neurodegenerative diseases and neural injury.

## References

[1] Wang CY, Kao TC, Lo WH, Yen GC (2011). Glycyrrhizic acid and 18β-glycyrrhetinic acid modulate lipopolysaccharide-induced inflammatory response by suppression of NF-κB through PI3K p110δ and p110γ inhibitions. J Agric Food Chem.

[2] Birari RB, Gupta S, Mohan CG, Bhutani KK (2011). Antiobesity and lipid lowering effects of Glycyrrhiza chalcones: experimental and computational studies. Phytomedicine.

[3] Kwon HJ, Kim HH, Ryu YB (2010). In vitro anti-rotavirus activity of polyphenol compounds isolated from the roots of *Glycyrrhiza uralensis*. Bioorg Med Chem.

[4] Wu TY, Khor TO, Saw CL (2011). Anti-inflammatory/anti-oxidative stress activities and differential regulation of Nrf2-mediated genes by non-polar fractions of tea *Chrysanthemum zawadskii* and licorice *Glycyrrhiza uralensis*. AAPS J.

[5] Kao TC, Shyu MH, Yen GC (2009). Neuroprotective effects of glycyrrhizic acid and 18beta-glycyrrhetinic acid in PC12 cells via modulation of the PI3K/Akt pathway. J Agric Food Chem.

[6] Wang W, Hu X, Zhao Z (2008). Antidepressant-like effects of liquiritin and isoliquiritin from *Glycyrrhiza uralensis* in the forced swimming test and tail suspension test in mice. Prog Neuropsychopharmacol Biol Psychiatry.

[7] Chen ZA, Wang JL, Liu RT (2009). Liquiritin potentiate neurite outgrowth induced by nerve growth factor in PC12 cells. Cytotechnology.

[8] Sun YX, Tang Y, Wu AL (2010). Neuroprotective effect of liquiritin against focal cerebral ischemia/reperfusion in mice via its antioxidant and antiapoptosis properties. J Asian Nat Prod Res.

[9] Traynelis SF, Wollmuth LP, McBain CJ (2010). Glutamate receptor ion channels: structure, regulation, and function. Pharmacol Rev.

[10] Nicholls DG (2004). Mitochondrial dysfunction and glutamate excitotoxicity studied in primary neuronal cultures. Curr Mol Med.

[11] Jang JY, Kim HN, Kim YR (2013). Hexane extract from *Polygonum multiflorum* attenuates glutamate-induced apoptosis in primary cultured cortical neurons. J Ethnopharmacol.

[12] Zhang M, Li J, Geng R (2013). The inhibition of ERK activation mediates the protection of necrostatin-1 on glutamate toxicity in HT-22 cells. Neurotox Res.

[13] Xia Z, Dickens M, Raingeaud J, Davis RJ, Greenberg ME (1995). Opposing effects of ERK and JNK-p38 MAP kinases on apoptosis. Science.

[14] Lin YL, Wang GJ, Huang CL (2009). *Ligusticum chuanxiong* as a potential neuroprotectant for preventing serum deprivation-induced apoptosis in rat pheochromocytoma cells: functional roles of mitogen-activated protein kinases. J Ethnopharmacol.

[15] Lou H, Fan P, Perez RG, Lou H (2011). Neuroprotective effects of linarin through activation of the PI3K/Akt pathway in amyloid-β-induced neuronal cell death. Bioorg Med Chem.

[16] Lu S, Lu C, Han Q (2011). Adipose-derived mesenchymal stem cells protect PC12 cells from glutamate excitotoxicity-induced apoptosis by upregulation of XIAP through PI3-K/Akt activation. Toxicology.

[17] Jin Y, Yan EZ, Fan Y (2007). Neuroprotection by sodium ferulate against glutamate-induced apoptosis is mediated by ERK and PI3 kinase pathways. Acta Pharmacol Sin.

[18] Mosmann T (1983). Rapid colorimetric assay for cellular growth and survival: application to proliferation and cytotoxicity assays. J Immunol Methods.

[19] Cossarizza A, Baccarani-Contri M, Kalashnikova G, Franceschi C (1993). A new method for the cytofluorimetric analysis of mitochondrial membrane potential using the J-aggregate forming lipophilic cation 5,5′,6,6′-tetrachloro-1,1′,3,3′-tetraethylbenzimidazolcarbocyanine iodide (JC-1). Biochem Biophys Res Commun.

[20] Yang CL, Chik SC, Li JC, Cheung BK, Lau AS (2009). Identification of the bioactive constituent and its mechanisms of action in mediating the anti-inflammatory effects of black cohosh and related Cimicifuga species on human primary blood macrophages. J Med Chem.

[21] Lee CS, Kim YJ, Lee MS, Han ES, Lee SJ (2008). 18beta-Glycyrrhetinic acid induces apoptotic cell death in SiHa cells and exhibits a synergistic effect against antibiotic anti-cancer drug toxicity. Life Sci.

[22] Simon HU, Haj-Yehia A, Levi-Schaffer F (2000). Role of reactive oxygen species (ROS) in apoptosis induction. Apoptosis.

[23] Ricci JE, Gottlieb RA, Green DR (2003). Caspase-mediated loss of mitochondrial function and generation of reactive oxygen species during apoptosis. J Cell Biol.

[24] Dejean LM, Martinez-Caballero S, Kinnally KW (2006). Is MAC the knife that cuts cytochrome c from mitochondria during apoptosis?. Cell Death Differ.

[25] Dudek H, Datta SR, Franke TF (1997). Regulation of neuronal survival by the serine-threonine protein kinase Akt. Science.

[26] Dal-Cim T, Molz S, Egea J (2012). Guanosine protects human neuroblastoma SH-SY5Y cells against mitochondrial oxidative stress by inducing heme oxigenase-1 via PI3K/Akt/GSK-3β pathway. Neurochem Int.

[27] Ha T, Hua F, Liu X (2008). Lipopolysaccharide-induced myocardial protection against ischaemia/reperfusion injury is mediated through a PI3K/Akt-dependent mechanism. Cardiovasc Res.

[28] Ahmed NN, Grimes HL, Bellacosa A, Chan TO, Tsichlis PN (1997). Transduction of interleukin-2 antiapoptotic and proliferative signals via Akt protein kinase. Proc Natl Acad Sci USA.

[29] Chao DT, Korsmeyer SJ (1998). BCL-2 family: regulators of cell death. Annu Rev Immunol.

[30] Boucher MJ, Morisset J, Vachon PH, Reed JC, Lainé J, Rivard N (2000). MEK/ERK signaling pathway regulates the expression of Bcl-2, Bcl-X(L), and Mcl-1 and promotes survival of human pancreatic cancer cells. J Cell Biochem.

